# Deep multiple-instance learning for abnormal cell detection in cervical histopathology images

**DOI:** 10.1016/j.compbiomed.2021.104890

**Published:** 2021-09-28

**Authors:** Anabik Pal, Zhiyun Xue, Kanan Desai, Adekunbiola Aina F Banjo, Clement Akinfolarin Adepiti, L. Rodney Long, Mark Schiffman, Sameer Antani

**Affiliations:** a National Library of Medicine, National Institutes of Health, Bethesda, MD, USA; b National Cancer Institute, National Institutes of Health, Bethesda, MD, USA; c College of Medicine, Lagos University Teaching Hospital, Idi-araba, Lagos, Nigeria; d Obafemi Awolowo University Teaching Hospital, Osun State, Nigeria

**Keywords:** Cervical histopathology, High dimensional images, Multiple instance learning, Sparse attention, Dataset

## Abstract

Cervical cancer is a disease of significant concern affecting women’s health worldwide. Early detection of and treatment at the precancerous stage can help reduce mortality. High-grade cervical abnormalities and precancer are confirmed using microscopic analysis of cervical histopathology. However, manual analysis of cervical biopsy slides is time-consuming, needs expert pathologists, and suffers from reader variability errors. Prior work in the literature has suggested using automated image analysis algorithms for analyzing cervical histopathology images captured with the whole slide digital scanners (e.g., Aperio, Hamamatsu, etc.). However, whole-slide digital tissue scanners with good optical magnification and acceptable imaging quality are cost-prohibitive and difficult to acquire in low and middle-resource regions. Hence, the development of low-cost imaging systems and automated image analysis algorithms are of critical importance. Motivated by this, we conduct an experimental study to assess the feasibility of developing a low-cost diagnostic system with the H&E stained cervical tissue image analysis algorithm. In our imaging system, the image acquisition is performed by a smartphone affixing it on the top of a commonly available light microscope which magnifies the cervical tissues. The images are not captured in a constant optical magnification, and, unlike whole-slide scanners, our imaging system is unable to record the magnification. The images are mega-pixel images and are labeled based on the presence of abnormal cells. In our dataset, there are total 1331 (train: 846, validation: 116 test: 369) images. We formulate the classification task as a deep multiple instance learning problem and quantitatively evaluate the classification performance of four different types of multiple instance learning algorithms trained with five different architectures designed with varying instance sizes. Finally, we designed a sparse attention-based multiple instance learning framework that can produce a maximum of 84.55*%* classification accuracy on the test set.

## 1. Introduction

The etiology of cervical cancer is the same globally, as are the natural history steps in cervical carcinogenesis. Universally, cervical cancer is caused by persistent infections with up to a dozen types of human papillomavirus (HPV). HPV infections are among the most common sexually transmitted agents, and the great majority of infections even of the highest risk types are benign. This is due, in large part, to lifelong immune control (“clearance”). It is the absence of clearance, called persistence, that is associated with cervical precancer. Precancers are defined as high-grade intraepithelial lesions that are at substantial risk of developing into invasive cervical cancer, if undetected and untreated, and are confirmed using histopathological analysis. The closest diagnoses representing precancer are grade 3 cervical intraepithelial neoplasia (CIN3) and, uncommonly, adenocarcinoma in situ (AIS). The clinical features to be examined for differentiating high-grade CIN3 from normal tissue are cellular differentiation and maturation, organization, polarity, nuclear abnormalities, and mitotic activity as well as the proportion of epithelium showing disturbances of maturation and differentiation. Microscopic inspection of cervical tissue is the standard approach for examining above mentioned abnormalities. However, microscopic inspection of cervical tissue is tedious, error-prone, and demanding of expertise [1]. An additional source of error comes from the variation in staining and tissue processing. To advance the diagnostic system, nowadays, whole slide digital scanners (e.g., Aperio, Hamamatsu, etc.) are used to digitize the biopsy specimens. Biopsy sample inspection using the digitally scanned images is expected to reduce the opportunity for human errors that come from staining variation, and tissue variation as computer vision algorithms can be applied to perform image quality improvement [2–7]. However, the need for improved optics, advanced imaging sensors, computational processing equipment, and significant data storage make the high-quality digital scanners prohibitively expensive, particularly in low and middle-resource countries/settings. Hence, traditional optical microscopes are commonly used for reading tissue slides. Therefore, in the low and middle resource setting the actual need is to automate and improve standard optical microscope-based tissue inspection and reduce workload.

The development of a robust computer vision algorithm for analyzing camera images captured with optically magnified tissue is more challenging than the images that come from a whole slide scanner. These challenges come from the use of low-cost imaging sensors, lack of imaging standards, and human error in image acquisition. For these reasons, the available computational methods developed for analyzing digitally scanned tissue images may not be easily transferable to analyze optical microscopic images [8]. Therefore, there is a high research demand to develop an automated cervical tissue inspection system for smartphone camera-acquired tissue images magnified using a standard optical microscope.

In principle, the development of robust discriminative hand-crafted features (like [9–13]) to represent pathology and non-pathology regions of cervical tissue is challenging. The latest advances in data-driven supervised machine learning or deep learning (specifically deep Convolutional Neural Networks) techniques (i.e. [14–16]) can be employed for that. However, these approaches are data-hungry and computationally expensive. Their training data requirement depends on the complexity of the learnable function. Training a deep model with images containing finer details ensures better learning but it needs to process spatially high dimension images which increases the requirement of computing hardware. Hence, patch-based analysis has become a common practice for high dimensional image analysis problems such as digitally scanned whole slide biopsy image analysis [17], microscope assisted digital camera captured biopsy images [18], etc. In many cases, patch-based analysis approaches first segment the Region of Interest (RoI), and divide the segmented region into patches that are classified individually with a trained classifier. Finally, the patch classification predictions are aggregated for decision making [18]. These kinds of patch-based processing increase the requirement of annotation effort.

The overarching goal of our project is to develop a computational system for automatic cervical histopathology image analysis and characterize the disease condition. In this paper, we present an experimental study on cervical histopathology image classification based on the presence of abnormal cells. The cervical histopathology images available in our data set are acquired using a smartphone camera affixed to the eyepiece of the microscope. As they are spatially high-dimensional, the development of deep multiple instance learning (MIL) [19] based framework is an effective approach. Deep MIL is an advancement to train a deep Convolutional Neural Networks for high-dimensional data. In a MIL for image analysis context, the bag represents an image and every bag consists of multiple patches called the instances. Developing an effective deep MIL algorithm for image classification needs both engineering and algorithmic efforts. The engineering effort refers to performing empirical analysis to build a suitable deep MIL architecture. Note that, no state-of-the-art MIL architecture for histopathology image analysis is available for general use, and the MIL network architectures vary based on the imaging modality, the object size, magnification, etc. On the other hand, the algorithmic effort requires deciding instance sampling and aggregating procedure. A digital image can be divided into regularly spaced either overlapping or non-overlapping patches (i.e. instances). However, to minimize the computational burden and error due to the excessively high number of non-informative patches, a selection of non-overlapping patches from the RoI is intended. Note that the RoI determination needs domain-specific heuristics or experts’ annotation. Unfortunately, neither is available for our dataset. The instance aggregation is of mainly two types- (i) aggregation of instance embedding (feature) and (ii) aggregation of instance predictions. The use of statistical or attention-based pooling is commonly used for both instance prediction aggregation or instance embedding aggregation [20,21].

In summary, this paper develops a sparse attention-based deep MIL framework for cervical histopathological image analysis. The sparsity in the attention weights aims to automatically discard non-informative instances (i.e. instances which are not lying in the anatomical regions to be inspected) and thus increases the probability for the probable positive instances. The key contributions of this paper are as follows:

- We believe that this is the first effort that utilizes sparse attention for instance aggregation in a deep multiple-instance learning framework. We analyze the algorithm’s performance and effectiveness for cervical histopathology image classification using several architectures.
- To the best of our knowledge this work is the first attempt toward developing an automated low-cost cervical histopathology image analysis system. The development of a H&E stained cervical histopathology image dataset for conducting this research is another by-product of this research.

The organization of the paper is as follows. Section 2 discusses the available literature on cervical tissue image analysis and the medical image analysis with weak labels. The imaging protocol, methodological background, and the considered Deep MIL frameworks are explained in Section 3. The experimental protocol is given in Section 4. The experimental results are discussed in Section 5. Finally, we conclude with Section 6.

## 2. Related literature

### 2.1. Cervical tissue image analysis

The overarching goal of cervical histopathology image analysis is to confirm and stage cervical neoplasia and characterize the disease condition. Several published works describe methods for automatically classifying these images into one of the CIN grades [2–7]. All approaches use sub-regions of the whole slide scanned images where expert annotations limit machine processing to tissue regions where the disease might exist. All methods have several common steps. First, the methods segment (interactively [2] or automatically [3–6]) the epithelium layer from the stroma, and then analyze the segmented region using a combination of traditional computer vision and machine learning approaches [2–5], or more recently, they use deep learning [6]. A brief description of the available works is given in the following paragraphs.

De et al. [2]. attempted to develop an automated image analysis algorithm for classifying the images into one of the four classes (Normal, CIN1, CIN2, and CIN3 grades). This method comprises the following steps. First, the squamous epithelium region is manually segmented by an expert pathologist. Then medial axis detection, vertical segments generation, feature extraction (texture, triangle, and profile-based correlation feature), classification (with LDA classifier), and finally fusion of the CIN grades from each vertical segment are conducted for producing classification output. The algorithm was tested on a dataset containing 61 cervical histology images. The experimental results show that the proposed algorithm improves the classification performance by a huge margin (15.5%) using manually segmented regions, instead of a global feature for the entire image.

Wang et al. [3]. introduce an automated system for analyzing WSI cervical histological digital slides to detect CIN. Their approach segments the squamous epithelium and then detects and grades CIN by analyzing the segmented region with domain-specific features like nuclei structure, morphology, etc. The high spatial dimension histology images are segmented with a region-based multi-resolution texture classification method. First, an initial block-based coarse segmentation is performed at a low resolution (2X), and then the boundary blocks are refined at a higher resolution for getting fine-grained segmentation output. A support vector machine-based classifier is used for performing the texture classification task. Thirty-one (31) digital slides that are scanned at 40X objective magnification are used in this research. The method achieves 94.25% segmentation accuracy and a maximum of 94.87% CIN diagnosis performance.

Keenan et al. [5] use hand-crafted geometrical features (mean area, the mean edge length, and the occurrence per unit area) of Delaunay triangles formed with vertices as the centers of all nuclei within the epithelium. These features are used for discriminant analysis for CIN grading. A dataset of 230 (normal: 30, koilocytosis: 46, CIN1: 52, CIN2: 56, and CIN3:46) digital images, annotated by an expert gynecological pathologist, is used for evaluation. The methods result in 98.7% accuracy in discriminating between normal and CIN3, 76.5% accuracy in discriminating koilocytosis and CIN1, whereas, only 62.3% accuracy in discriminating between CIN cases. The reported misclassification rate is highest for CIN2.

An automated, localized, fusion-based approach is proposed in Ref. [6] for analyzing digitized histology images for classifying uterine cervix squamous epithelium into Normal, CIN1, CIN2, and CIN3 grades. In summary, the key components of the proposed system include the following: detection of the medial axis of the segmented epithelium region, division of the segmented image into 10 vertical segments orthogonal to the medial axis, feature extraction from each of the vertical segments, classification of each segment into one of the CIN grades, and then, fusing CIN grades from every ten vertical segments. A dataset containing 118 manually segmented epithelial regions from a selected collection of whole-slide histology images is used to evaluate the method. Based on a leave-one-out approach for classifier training and testing, exact grade CIN accuracies of 81.29% and 88.98% were achieved for the individual vertical segment and epithelium whole-image classification, respectively.

A multilayer hidden conditional random fields (MHCRFs)-based cervical histopathology image classification (CHIC) model is proposed in Ref. [7] to classify different stages of cervical cancer using a weakly supervised learning. Firstly, hand-crafted color and texture features are combined with deep features obtained from the pre-trained model. The combined feature set obtained from the histopathological image patches is used to train 3 classifiers (artificial neural network, support vector machine, and random forest) for predicting patch-level classification probabilities. The effective classifiers are selected to generate unary and binary potentials. At last, using the generated potentials, the final image-level classification results are predicted by our MHCRF model. The trained model achieves an overall accuracy of around 77.32% on six (6) real cervical histopathological image datasets with more than 600 immuno-histochemical (IHC) stained samples.

A computer-aided decision support system (CADSS) is developed in Ref. [4] to identify abnormalities and quantify cancer grading from histopathological images. The proposed system is a multi-step approach including image acquisition, pre-processing, segmentation, feature extraction, classification, grading, and disease identification. Digital images of spatial dimension 4080 × 3072 are captured with a charge-coupled device (CCD) camera integrated with the microscope. Then, the captured image is passed through a noise removal module to reduce the noise and improve the segmentation performance performed in the next step. Several hand-crafted morphological and texture features are extracted from the segmented epithelium region for classification. The method is evaluated on a dataset of 475 cervical biopsy images. Their best specificity and sensitivity scores are 0.97 and 0.9975, respectively.

In summary, cervical histopathology image analysis is an insufficiently explored research avenue. Analysis of biopsy images obtained from whole slide scanners is considered for most research. Several primitive computer vision algorithms based on hand-crafted color, texture, and geometrical features are typically employed for the epithelium region analysis. However, the performance of these methods is highly dependent on the ROI segmentation and strong labels. Moreover, correct computation of geometric features remains challenging in the presence of image artifacts or tissue artifacts. Despite the algorithmic limitation, the present imaging system brings additional challenges in terms of variability, and unavailability of magnification. Moreover, we have annotation only at the image level; ROIs are not labeled. According to our survey, the commonly used algorithms are not directly applicable for analyzing these histopathological images. Note that conventional Papanicolaou smear (CPS) and liquid-based cytology (LBC) are performed for detecting abnormal cells in cervical tissue which leads towards the development of several image analysis algorithms [22–28]. On contrary, the overarching goal of our research is to analyze smartphone captured H&E stained cervical histopathology images and characterize disease conditions automatically. However, in this paper, we aim to classify an image based on the presence of abnormal cell(s).

### 2.2. Medical image analysis with weak labels

Deep learning algorithms are increasingly popular in research and practical applications due to their ability to automatically feature learning from the training data. However, the development of a robust deep model is challenging as it is data-hungry, needs strongly labeled and class-balanced data. Hence, the real need is to develop a deep model from a low volume, class-imbalanced dataset having noisy, partial, and weak annotations. Several annotation efficient deep learning algorithms are proposed for medical image analysis [29,30] with self-supervised learning [31], semi-supervised learning [32], deep metric learning [33], multiple instance learning [34] etc. For a histopathological image analysis task, the labels are often not available at the desired granularity due to their high spatial dimension [35]. Hence, the use of multiple instance learning is common for segmentation [36], detection of abnormality [37], and disease characterization [21]. According to our survey, no generic architecture is available for multiple instance learning-based histopathological image analysis. The multiple instance learning algorithms are varied based on the instance size assumption and the instance aggregation strategy [20,21,38–41].

### 2.3. Deep learning for H&E stained tissue image analysis

Nowadays deep learning algorithms especially variants of deep convolutional neural networks are widely used for analyzing H&E stained tissue image analysis for cancer screening [42–44], cancer grading [45,46], cancer type prediction [47], tissue quantification and genomic correlations [48], tissue segmentation [49,50], gland segmentation [51,51,52], skin layer segmentation [53], nuclei segmentation [54–56], nerves and blood vessels [57] etc. However, according to our survey, the potential advantage of using advanced deep learning algorithms is not well explored for the cervical histopathology image analysis task.

## 3. Material and methods

### 3.1. Data collection and imaging protocol

Our data was acquired during an epidemiological study on cervical screening in Nigeria which included 9407 female subjects (aged 30–49 years) residing in the catchment area of Obafemi Awolowo University Teaching Hospitals Complex (OAUTHC) in Ile-Ife, Nigeria. The research study is approved by National Cancer Institute (iRIS reference number: 376424; IRB number: 09CN045; Version date: 12/20/2017) and OAUTHC ethical Institutional Review Boards (Protocol number: ERC/2016/05/08 and ERC/2018/09/10; Registration number international: IRB/IEC/0004553, national: NHREC/27/02/2009a). Multiple biopsies including punch biopsies, endocervical curettage (ECC) sampling, and large loop excision of the transformation zone (LLETZ) biopsies were collected from every subject depending on the clinical indication. Only those subjects whose cervix developed acetowhite reaction following application of 5% acetic acid (confirmed by an expert gynecologic oncologist with colposcopic examination) were considered for Endocervical Curettage (ECC) tissue specimen collection. The collected tissues were sliced and then processed with H&E staining. One or more slides were prepared per biopsy specimen collected depending on the type of the sample (i.e. typically one slide is prepared for an ECC sample versus up to 20 slides could be prepared for the LLETZ specimen depending on the size of the excised tissue and processing).

**Fig. 1.**
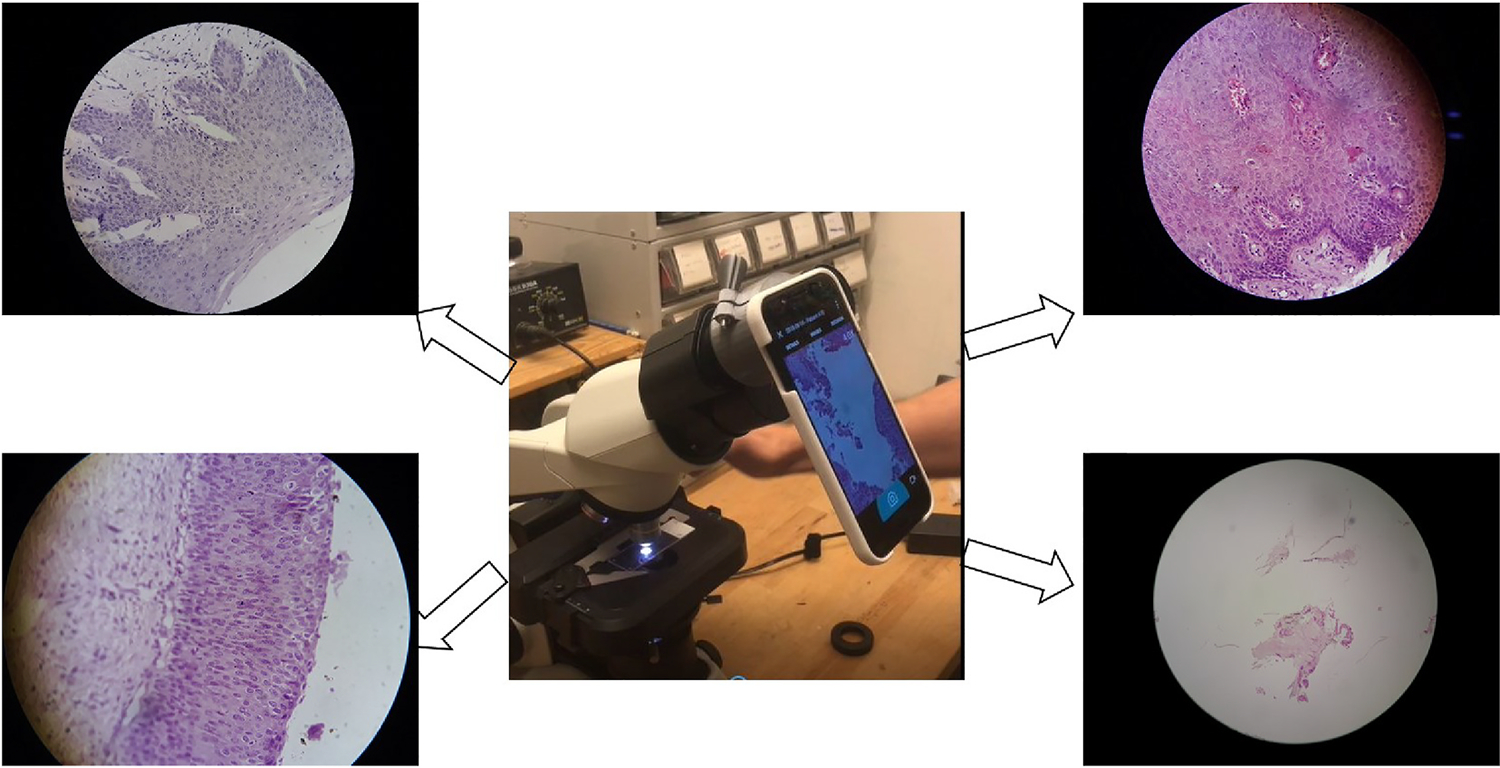
Image acquisition. Samsung J5 Smartphone connected to the optical microscope via an adapter (center, credit: MobileODT, Israel) along with different cervical histopathology images captured using it. Among four different sample images except bottom-left image, all three cover the full tissue region. There is also variety in magnification among the images which cover the tissue specimen completely.

To reduce the imaging cost, the prepared glass slides were imaged with a Samsung J5 smartphone (MobileODT, Israel) affixed to the eyepiece of an optical microscope (see Fig. 1) using a specialized adapter. The smartphone camera captures the photographs of the cervical tissue kept under the microscope with chosen magnification. An image captured with higher magnification can capture the tissue anatomy better way but cannot include the available tissue fully. On the other hand, an image captured with lower magnification can cover the tissue fully but the quality of the cell/tissue boundaries are degraded. The images were primarily captured for clinical record keeping, hence no standardization was followed to maintain the magnification. For the present research, we select only those images which cover the tissue fully. However, we observe that even the images which contain the tissue fully have variability in magnification.

### 3.2. Deep convolutional neural network

Deep Convolutional Neural Networks (DCNN) are the current state-of-the-art approach for image classification problems. Briefly, the DCNN models contain multi-stage data processing units called layers [14,15] and the organization of these layers in a DCNN model is called the network architecture (or, architecture). In a DCNN, some layers have learnable parameters (called parametric layers) and others do not have (called non-parametric layers). To develop an image classifier, firstly, a multi-layered DCNN architecture is designed and then the parameters of DCNN (for the parametric layers) are finalized with an iterative approach called model training. A loss function is designed to compute the deviation between the predicted and the ground-truth class probability vectors for a particular step in training. The computed loss act as a supervisory signal to update the DCNN parameters. The learning (or training) procedure stops when a predefined condition is fulfilled. Thus a DCNN trained with supervised training can produce discriminative features and relieves researchers in designing domain-specific hand-crafted discriminative feature representation of the images. However, the development of an effective DCNN model for an image classification task is challenging as it needs empirical study. The computational cost for model training depends on the training image size, the number of training images, and the number of network parameters.

For the present image classification problem, the development of a well-generalized DCNN model is challenging due to the high spatial dimension of the histopathology images. Generally speaking, to keep finer image details of the cervical histopathology, we have to design a network with more layers than the traditional classification networks that are trained on images with significantly smaller dimensions [14, 15]. Increasing the number of layers will increase the number of model parameters. This will place a significantly high computational burden for model training. Moreover, to have a generalized model we need to train the DCNN with a huge volume of training data.

### 3.3. Multiple instance learning

Multiple Instance Learning (MIL) is an intelligent supervised learning algorithm for learning from weakly annotated data. In MIL, a bag represents individual data elements, and every bag consists of multiple instances of that element [58–60]. In binary classification, every instance must have either a positive or a negative label. In the basic MIL algorithm, a bag is tagged positive if *at least one* positive instance is available in it and a bag is negative if and only if *all* of its instances are negative [61]. Therefore, this type of learning belongs to the class of weakly supervised learning algorithms since the positive class usually contains an imprecise signal. The MIL approach can be divided into the following three subcategories - (a) Bag-based: where feature embedding of all instances in a bag are analyzed for detecting dissimilarities among bags; (b) Instance-based: where the positive class probability of the individual instances are computed and then aggregated for bag label prediction; and, (c) Embedding-based: where feature embedding for all instances are aggregated and then fed to the classifier for bag label prediction [62].

### 3.4. Deep multiple instance learning

Deep Multiple Instance Learning can be considered as an advanced deep model training strategy. Intuitively, the bag represents a data element that can be divided into multiple chunks called instances. The MIL algorithms process every instance individually with DCNN and finally aggregate the results to produce the intended class label prediction. It is assumed that preservation of arrangement or order of the instances is not required. Hence, the DCNN model’s parameters are shared among instances that helps to reduce model parameters. The effectiveness of a deep MIL for image classification depends on three factors. The first important factor is how to sample patches (i.e. instances) from an image. The patch size is decided based on the object’s spatial dimension and both non-overlapping and overlapping patch sampling can be considered. Availability of prior information about probable abnormal regions can optimize the computational burden as well as reduce error due to consideration of non-informative negative instances. The next important factor is the deep architecture to be used for processing the instances. Note that unlike, other standard whole image classification DCNN models, no well-accepted general-purpose MIL architecture is available. Finally, instance aggregation strategy is an important factor for model training. DCNN can be designed to produce either the patch embeddings or the prediction scores. A MIL algorithm that aggregates the embeddings is called Embedding-based MIL (EMIL) and a MIL algorithm that aggregates the instance predictions is called Instance-based MIL (IMIL).

The instance aggregation (or MIL pooling) function in multiple instance learning will have to be permutation invariant [61,62] i.e. instance order will not change the aggregated output. Any statistical pooling operation (like max-pooling, an average of top-K pooling) can be considered for this purpose [20,39,40] as they are permutation invariant. However, theoretically, these MIL pooling functions need to have prior information about the number of probable positive instances in the abnormal histopathology images and domain knowledge can be employed to select only probable instances for aggregation with top-K mean pooling [38]. Attention-based MIL aggregation is an effective choice when domain knowledge is not available. In attention-based instance aggregation, instances are weighted for aggregation. A two-layer self-attention module is trained to produce a weight vector which is further processed with a probability transformation function to convert the vector into a probability distribution [21]. In this regard, the Softmax function is a widely accepted probability transformation function. The mathematical expression of the Softmax function is as below:

(1) $$\text{softmax}\left(v_{i}\right)=\frac{\text{exp}\left(v_{i}\right)}{\sum_{j=1}^{L}\text{exp}\left(v_{j}\right)}$$

where the length of the weight vector is L and ith entry in the vector is denoted by $v_{i}$. However, the use of the Softmax attention for probability transformation has limitations as the output vector contains non-zero (whatever small value may be) value for all positions in the transformed vector^1^ so it will consider all instances for aggregation. Hence, in this paper, we design and experiment with sparse attention-based MIL aggregation.

### 3.5. Sparse attention based MIL aggregation

Sparse attention map generation is an active research area in machine learning [63–65]. In this paper, we employ following two different ways for that: regularization and the use a function that converts a linear vector into a sparse probability distribution. L0 norm Regularization [66] and Maximum Entropy Regularization [67] are effective regularizer to produce sparse linear vector and ‘Sparsemax’ function [65] is an advancement of ‘Softmax’ function which convert a linear vector into sparse probabilities. Although L0 normalization [67], Maximum Entropy Regularization [67], Sparsemax function [68,69] are utilized in the different contexts of machine learning problems, we believe that this is the first attempt where it is employed for MIL aggregation. A brief description of L0 Norm Regularization, Maximum Entropy Regularization, and Sparsemax function are given below.

#### 3.5.1. L0 Norm regularization

L0 Norm penalizes the number of non-zero entries and thus brings sparsity in a vector [66]. Let V denotes the N dimensional output vector obtained from the considered layer of a deep network. Then to regularize the layer with L0 norm, the network’s objective function will add a penalization term of $R_{L0}=\lambda_{L0}*\sum_{i=1}^{N}I\left[v_{i}\neq 0\right]$, where I[x] is a indicator function and is 1 when x≠0 and 0 otherwise; $\lambda_{L0}$ is a hyper-parameter which serve as the weighting factor for the regularization.

#### 3.5.2. Maximum Entropy Regularization

Maximum Entropy Regularization (MER) decreases the entropy of the elements in a probability vector and thus provide higher probability values to some positions [67]. Let V denotes the N dimensional probability transformed output vector obtained from the considered layer of a deep network. Then to regularize the layer with MER, the network’s objective function will add a penalization term of ${R_{\text{mer}}=\lambda }_{\text{mer}}*\sum_{i=1}^{N}v_{i}\;\text{log}\;v_{i}$; where $\lambda_{\text{mer}}$ is a hyper-parameter which serve as the weighting factor for the regularization.

#### 3.5.3. Sparsemax function

The sparsemax function converts a real valued vector into sparse probability distribution [65]. Mathematically, the sparsemax function generates the Euclidean projection of the input vector v onto the probability simplex. Let $v_{\left(1\right)}\geq v_{\left(2\right)}\geq \ldots \geq v_{\left(L\right)}$ are sorted co-ordinates of v; $l(v)=max\left\{l\in [L]|1+lv_{\left(l\right)}>\sum_{j\leq l}v_{\left(l\right)}\right\}$ then the mathematical expression of the sparsemax function is as below:

(2) $$\text{sparsemax}\left(v_{i}\right)=\text{max}\left(v_{i}-Th_{v},0\right);\;\text{where}\;Th_{v}=\frac{\sum_{j\leq l_{v}}v_{\left(j\right)}-1}{l(v)}$$

### 3.6. The MIL framework

**Fig. 2.**
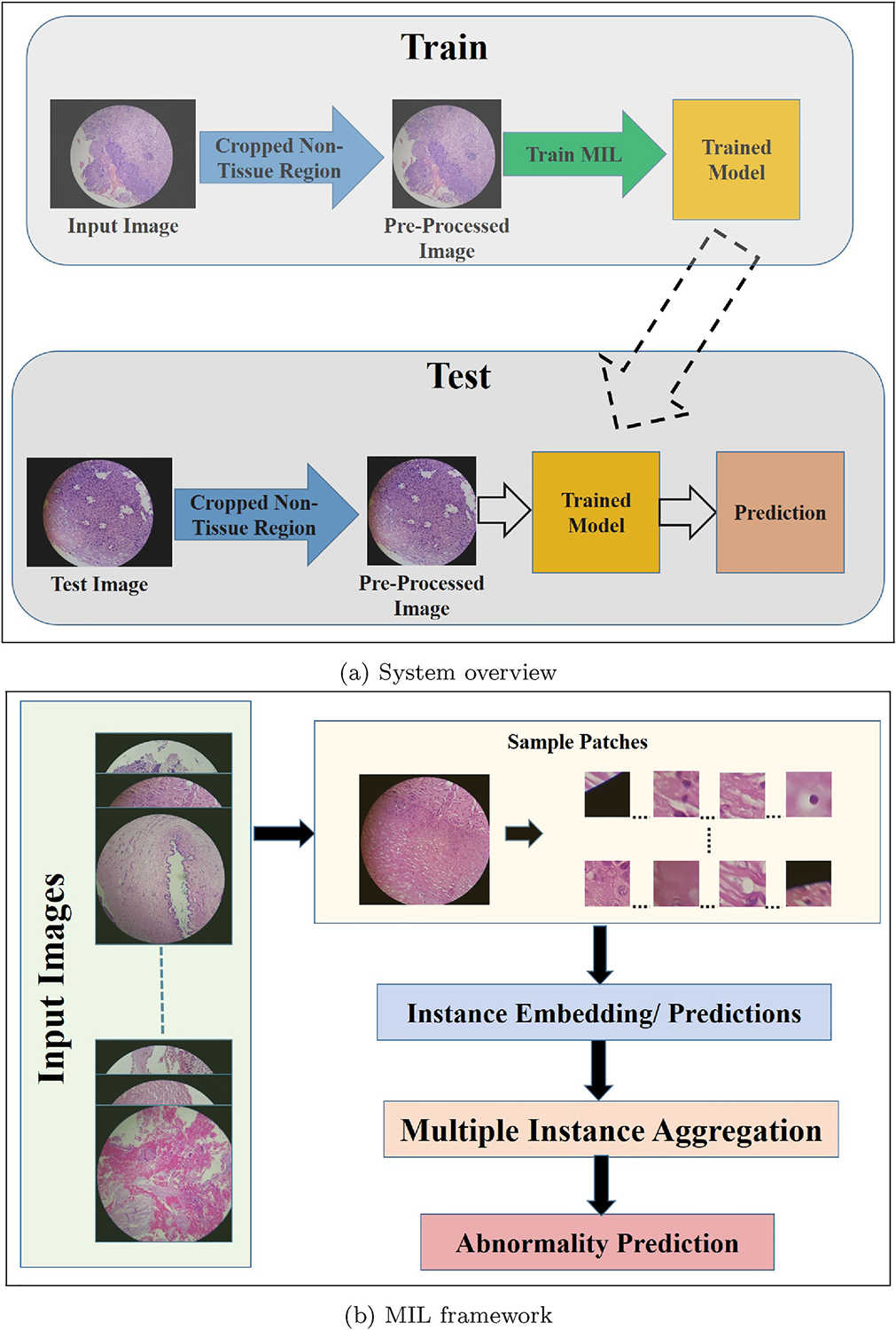
Description of the system.

A block diagram of the system framework is shown in Fig. 2a. The original spatial dimension of the images was 4128 pixel × 3096 pixel. As the tissue region appears as a circular region with a maximum diameter of 3096 pixels, a heuristic algorithm is employed in the pre-processing module to discard them. The pre-processing module reduces the spatial dimension of the images to 3096 × 3096. Next, the cropped images are used for model training; and for test images, the same pre-processing module is used to discard the black region and classification prediction is performed with the trained networks. In our framework individual images represent bags and all possible equal-sized, non-overlapping patches are considered as instances. The number of instances depends on the patch size. The pictorial diagram of the MIL modules used in our framework is shown in Fig. 2b. We experimented with both embedding aggregation (EMIL) and instance prediction aggregation (IMIL) and finally the performances are compared.

#### 3.6.1. Embedding-based MIL (EMIL)

**Fig. 3.**
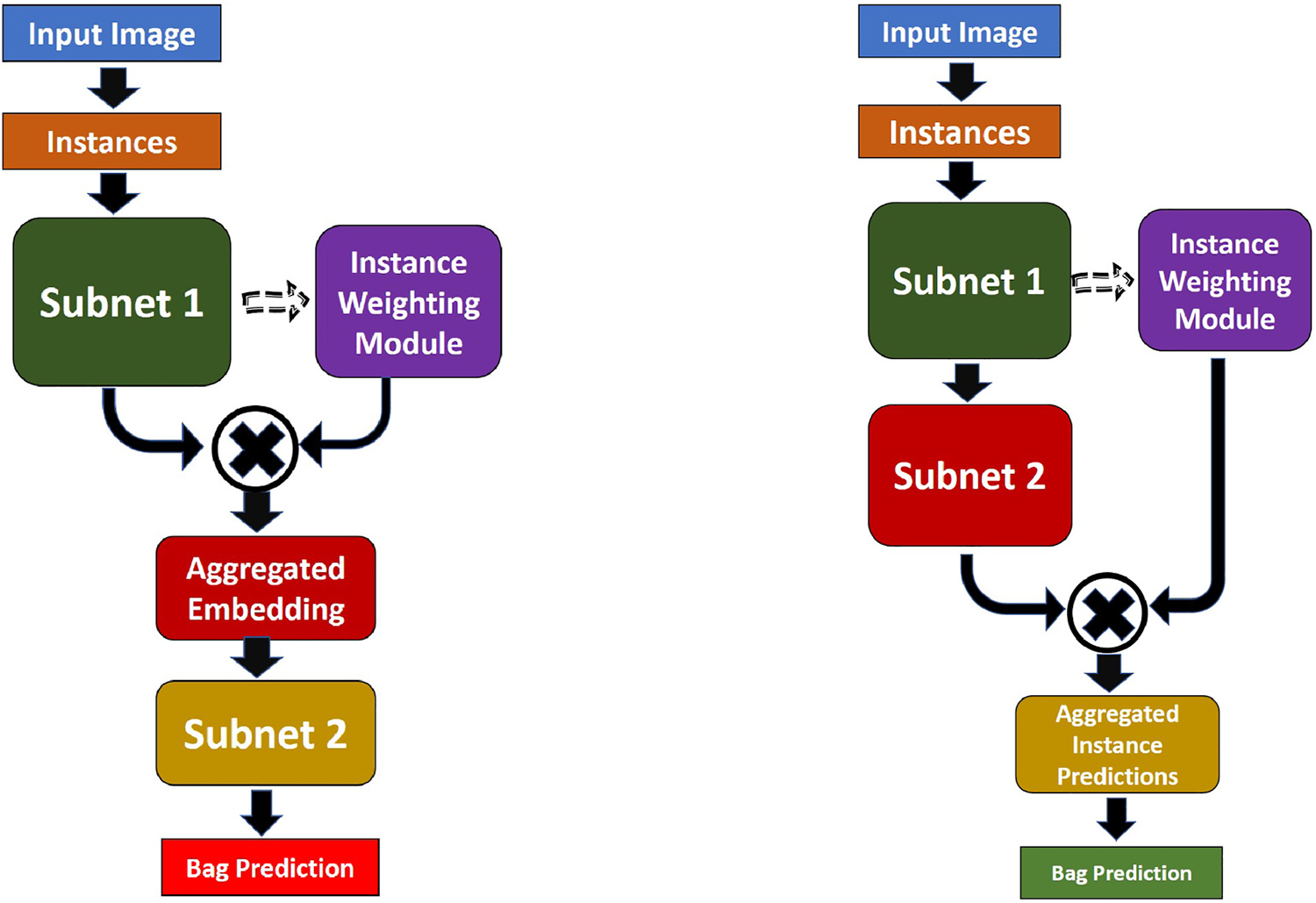
Block diagram of two different types of MIL.

The block diagram of the Embedding based MIL (EMIL) is shown in Fig. 3a. In EMIL, first, the input images are divided into instances called patches. Then process through Subnet 1 (the architecture is shown in Table 1). Then the embeddings are aggregated with an aggregation function. Then the output of the aggregated function is processed in Subnet 2 to produce bag level prediction. The Subnet 2 consists of four sequential layers (a) dropout layer with dropout probability 0.5, (b) dense layer with output node 256 with ReLU activation, (c) dropout layer with dropout probability 0.5, and finally (iv) dense layer with output node 256 with sigmoid activation.

#### 3.6.2. Instance-based MIL (IMIL)

The block diagram of the Instance-based MIL (IMIL) is shown in Fig. 3b. In IMIL, first, the input images are divided into instances called patches. Then process through Subnet 1 (the architecture is shown in Table 1). Then the embeddings are processed in Subnet 2 to produce instance-level predictions. Keeping similarity with EMIL, the Subnet 2 is built with four sequential layers (a) dropout layer with dropout probability 0.5, (b) dense layer with output node 256 with ReLU activation, (c) dropout layer with dropout probability 0.5, and finally, (iv) dense layer with output node 256 with sigmoid activation. Finally, the instances are aggregated to produce the bag-level prediction.

#### 3.6.3. Instance aggregation modules

In this paper, we analyze the performance of the above mentioned MIL frameworks with both **max-pooling-based** aggregation and **attention-based** aggregation. The attention-based aggregation learns a probability distribution function (i.e. weight for all instances and the sum of the weights = 1) for all instances. This mechanism uses a separate module that takes the output from Subnet1 and processes it with two layers. The first layer is dense (number of filters = 256) with the ‘tanh’ activation function and the second layer is a dense layer (number of filters = 1) with ‘sigmoid’ activation. Then for ‘Softmax’ attention, the instance weight vector obtained from the second dense layer is transformed into probability distribution with the ‘Softmax’ function. The dropout layers as described in Section 3.6.2 are used to regularize the network training.

In this paper, we propose **sparse attention-based** instance aggregation to increase the chance of discarding non-informative instances by setting their probability to 0. The novel instance aggregation technique is achieved with three modifications over ‘Softmax’ attention-based instance aggregation. First, penalization terms based on L0 norm regularization on the instance weight vector obtained from Subnet 2. Second, instead of applying the ‘Softmax’ probability transformation function on the instance weight vector, the ‘Sparsemax’ function is applied to generate a sparse probability vector. Finally, maximum entropy regularization (MER) is used to make the instance probability vector more sparse. Suppose, ${\text{Loss}}_{\text{no\_reg}}$ denotes MIL objective function when no regularizer is applied; $R_{L0}$ and $R_{\text{mer}}$ are penalization terms for regularization then the modified objective function $\left({\text{Loss}}_{\text{reg}}\right)$ is shown below:

(3) $${\text{Loss}}_{\text{reg}}={\text{Loss}}_{\text{no\_reg}}+R_{L0}+R_{\text{mer}}$$

## 4. Experimental protocol

### 4.1. Dataset

Our image dataset comprises 1331 images collected from 542 patients. The images were labeled by expert pathologists during photography. We have binary annotation i.e. normal or abnormal histopathology. The abnormal histopathology may belong to either of the following: Cervicitis (i.e. cervical inflammation), Low-grade Squamous Intra-epithelial Lesion (LSIL), High-grade Squamous Intra-epithelial Lesion, and Cancer. Note that the actual histopathology grade depends on the site where abnormal cells are developed.

We divide the image dataset into non-overlapping subsets: train set, validation set, and test set. An image-level random split may not bring all images of a patient into the same set. On the other hand, from our experience, a patient-level random split results in dramatically different normal and abnormal class ratios and it does not guarantee to have all variability of image magnifications in all partitions. Hence, manual effort is given for static data partition. We split the data patient-wise in a way such that every partition contains all variability of image magnification as well as have a closer normal and abnormal class ratio. A detailed description of the dataset in terms of the number of images for different classes is shown in Table 2.

### 4.2. Network training

We train the network with stochastic gradient descent (SGD) optimization algorithm. The images in the training set are randomly shuffled and inputted during training. The training is performed with batch size = 1 and online image augmentation (horizontal and vertical flipping) is used. The number of epochs is empirically decided and set to 50. The Keras [70] deep learning toolkit is used for model training and image prediction. Reverse class weighting is used to address the bag imbalance issue. The networks are trained with 2 GeForce RTX 2080 Ti GPUs installed with an Intel(R) Xeon(R) Gold 5218 CPU (@ 2.30 GHz).

### 4.3. Evaluation metrics

The present classification algorithm is evaluated with five evaluation metrics: (1) Area under curve of Receiver Operating Characteristic (ROC-AUC), (2) Accuracy (Acc), (3) Recall, (4) Precision, (5) F1-Score [71,72].

### 4.4. Baseline MIL networks

In this paper, we consider the following four different types of deep multiple instance learning approaches as baseline algorithms:

1. **EMIL_A**: Embedding-based MIL with Softmax attention aggregation. The architectural diagram is shown in Fig. 3a; and the architectural description is given in Section 3.6.1 and Section 3.6.3.
2. **EMIL_M**: Embedding-based MIL with max-pooling aggregation. It has the same architecture as EMIL_A but the instance aggregation module does not have any learning parameter as max-pooling is performed on every dimension of the embeddings.
3. **IMIL_A**: Instance-based MIL with Softmax attention aggregation. The architectural diagram is shown in Fig. 3b; and the architectural description is given in Section 3.6.2 and Section 3.6.3.
4. **IMIL_M**: Instance-based MIL with max-pooling aggregation. It has the same architecture as IMIL_A but the instance aggregation module does not have any learning parameter as max-pooling is performed on the instance predictions.

We compare the performance of the developed **IMIL_SA** (Instance-based MIL with sparse attention aggregation) with IMIL_A. For a fair comparison, the architecture of the IMIL_SA is kept same as IMIL_A.

## 5. Experimental results

**Fig. 4.**
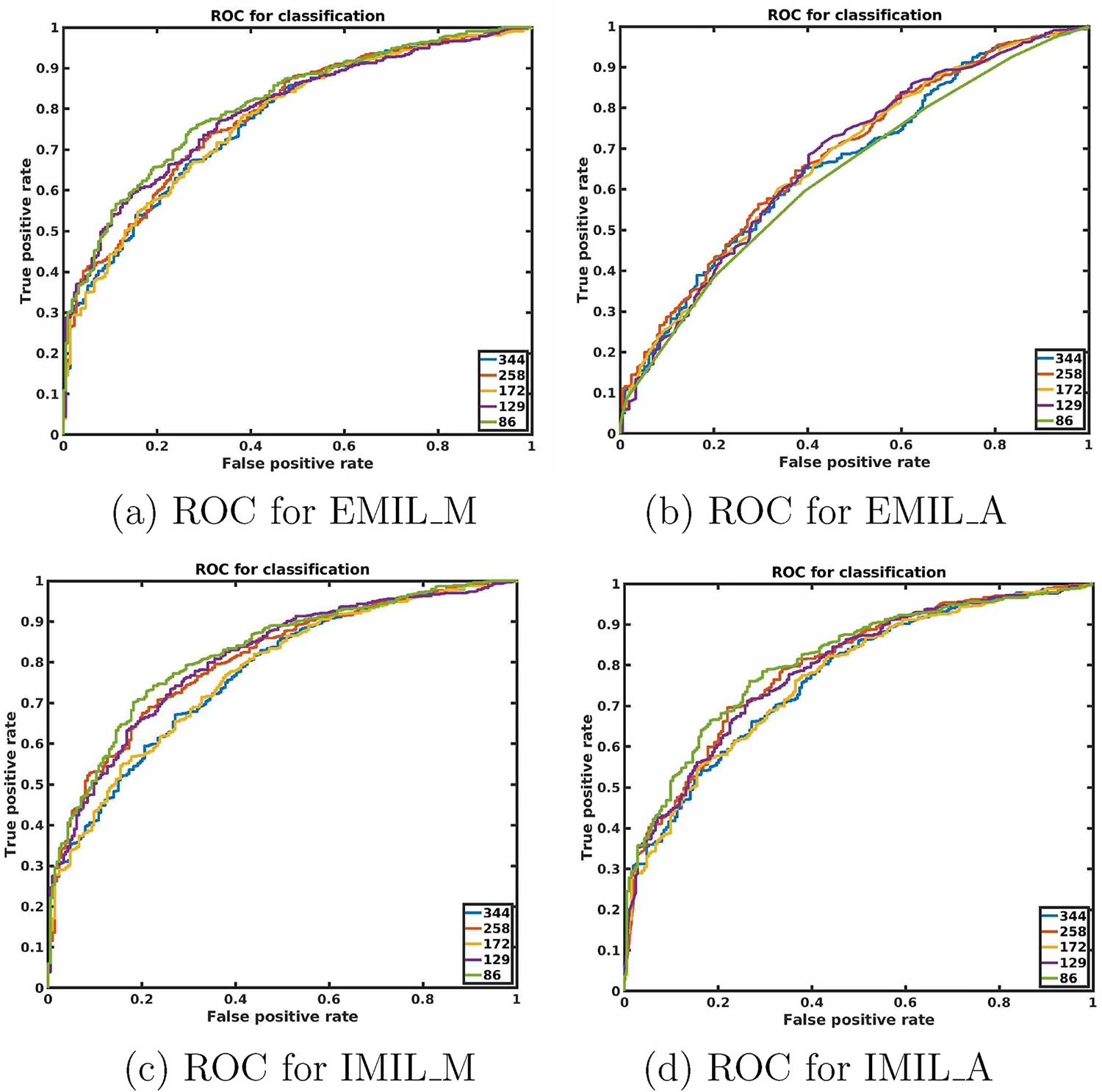
ROC curves for all competing methods.

We evaluate the performance of four different types of MIL algorithms presented in Section 4.4 and for every algorithm, we use five different sized patches. Note that a higher size patch may not capture abnormality if the image magnification is low. On the other hand, a lower size patch increases the computation burden, and may not capture abnormality if the image magnification is high. We vary the learning rate among (0.001, 0.005, 0.0001, 0.0005, 0.00001, 0.00005, 0.000001, 0.000005). The receiver operating curves (ROC curves) for best performing models are given in Fig. 4 and quantitative evaluation performance are reported in Table 3. The last row of Table 3 contains the performance of ResNet-50 a well-known classification network. According to the experimental results, deep multiple instance learning is more suitable than training classification networks for the present application. There is no noticeable performance gap with changing patch size for IMIL_A, IMIL_M, EMIL_M. Interestingly, best-performing models for different instance sizes are not produced with the same learning rate. The attention-based EMIL produces the worst prediction model and this is not recommended for the present task. We find the best accuracy (ACC) when IMIL_A is trained with an instance size of 129 pixel.

**Fig. 5.**
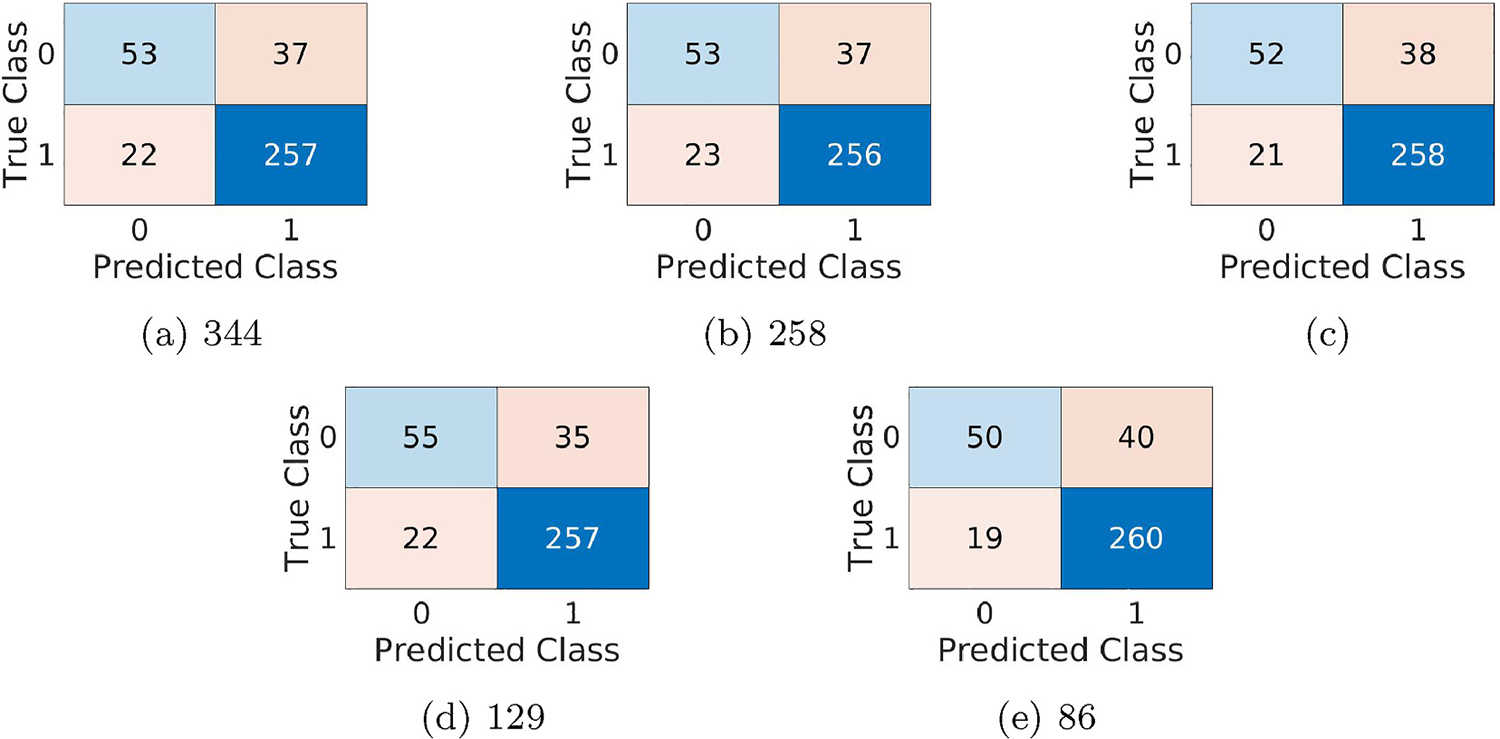
Confusion Matrices for the developed IMIL_SA algorithms. The captions denote the Instance/patch sizes of the IMIL_SA architectures.

**Fig. 6.**
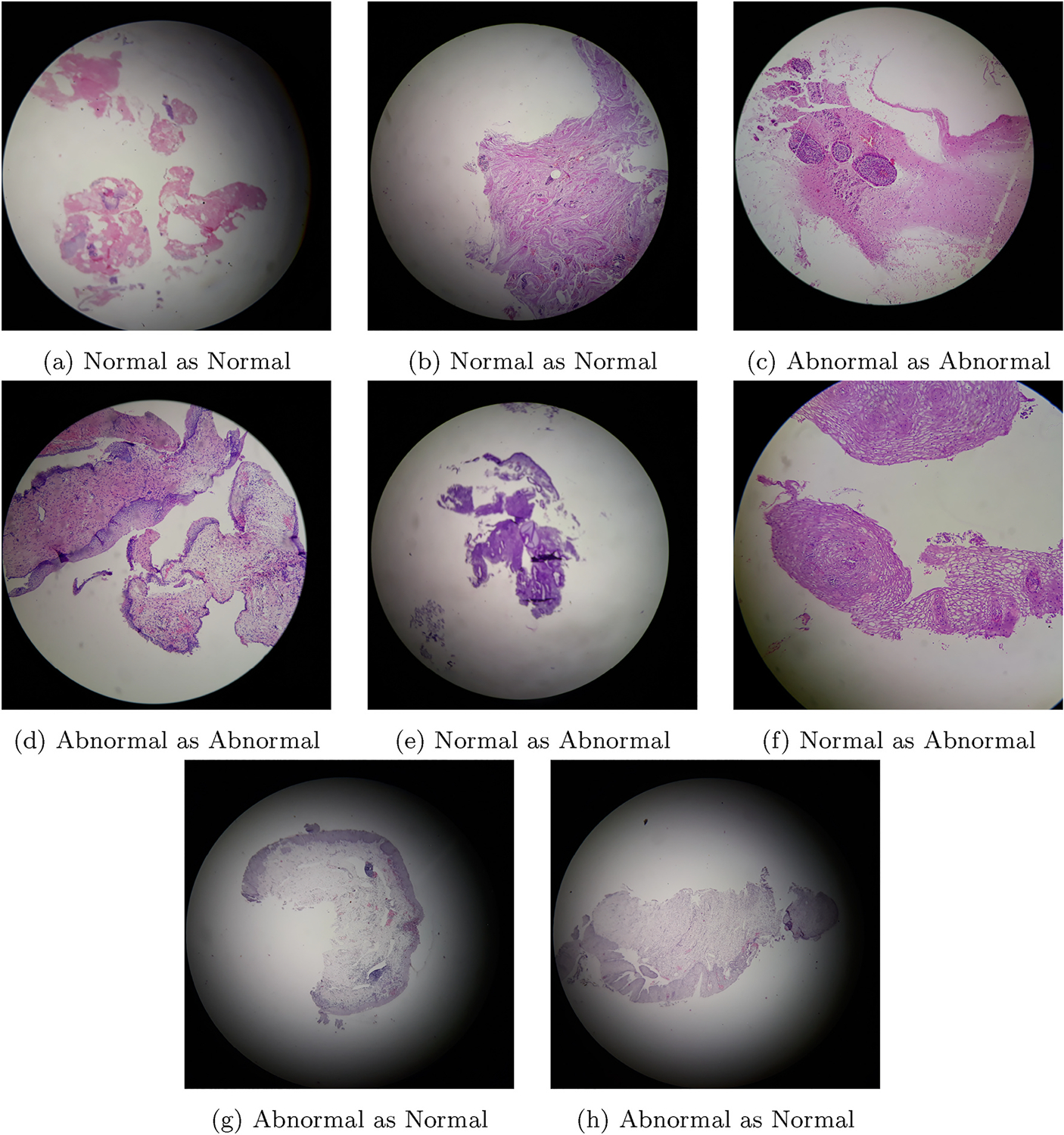
Images, their ground truth and predictions by IMIL_SA.

Our next interesting analysis is whether sparse attention can improve Softmax attention-based multiple instance learning. As only for instance based MIL attention based aggregation is capable of improving accuracy than max-pooling based aggregation, we consider performance analysis of Sparse Attention-based IMIL (IMIL_SA). The important parameters of IMIL_SA are patch size, $\lambda_{L0}$ and $\lambda_{\text{mer}}$. Hence, we evaluate the performance variation with different combinations of these parameters. The area under the curve (AUC) of the receiver operating curve (ROC) for varying patch size, $\lambda_{L0}$ and $\lambda_{\text{mer}}$ are shown in Table 4. Note that for a particular patch size, we choose the same learning rate where IMIL_A is performing best and vary the value of $\lambda_{L0}$ and $\lambda_{\text{mer}}$. The performance of the best performing model for a network is shown in Table 5 and the confusion matrices are shown in Fig. 5. Comparing the performance of IMIL_A presented in Table 3, and the performance of IMIL_SA presented in Table 4, we see that sparse attention improves the accuracy (ACC) for all MIL networks (varied based on instance size). The best-performing model is received when the instance size is 129. Some example images with their expert annotated class labels and the predicted class labels are given in Fig. 6.

## 6. Conclusion and scope of future work

This paper highlights the importance of developing an imaging algorithm for analyzing cervical histopathology images captured with low-cost imaging equipment. An image dataset consisting of cervical histopathology images captured with a microscope-assisted smartphone that has variability in magnification is used for experimental validation. It is worth mentioning that typical cervical precancer rates are 1–1.5%, so it would require screening and biopsying over 10, 000 subjects to generate approximately 150 cases. Such extensive studies are conducted infrequently hence utilization of biopsy images collected during a population study in Nigeria is a pioneering initiative to advance the related field. In this paper, we aim to classify an input image based on the presence of abnormal cells in it. The images are coarsely annotated i.e. only image-level labeling is available. We design a deep neural architecture and train the network with multiple instance learning (MIL) algorithms and make the advancement of MIL algorithm through sparse attention. The experimental results show that MIL is an efficient candidate to produce promising accuracy and the developed sparse attention incorporated instance-based MIL improves the performance than traditional Softmax attention incorporated instance-based MIL. We hope that the present research will attract the clinical community to make an organized research effort to build an automated cervical histopathology assessment system with low-cost imaging equipment.

Based on our belief and knowledge in the machine learning domain, we recommend the two immediate research steps for advancing our research one step forward towards the overarching clinical goal. First, we find that the error in the MIL comes due to the mathematical limitation of MIL when it has to process a huge number of negative instances. Note that we tried with several thresholding techniques to discard non-issue regions however no approach appears to work consistently. We see that there is color variation in the non-tissue regions. Hence, we need a separate computational model to select only informative patches (i.e. patches from the anatomical region to be inspected) from the biopsy. Development of segmentation annotation for tissue region or part of the tissue region where abnormality may appear will be useful in this regard. Availability of segmentation annotation helps to train a segmentation network that guides to sample patches from the segmented region for MIL. Second, research effort will be given for image standardization. The image dataset will be captured in a way such that all have the same optical magnification as well as the images will have the necessary quality for automated analysis. Interestingly, the prediction performance of a deep model depends on the training data samples. Hence, altering the training dataset can change the performance. In a general sense, exclusion of data from the training set increases the chance of performance drop and inclusion of more data in the training set increases the chance of performance boosting. Hence, increasing the training data by collecting tissue samples from the women will be an important step to advance the related study.

## Figures and Tables

**Table 1:** Architecture details of subnet 1.

Layer Name	No of Filter	Window size	Activation
Convolution 2D	16	(5,5)	ReLU
Max-Pooling 2D	–	(3, 3)	–
Convolution 2D	32	(3, 3)	ReLU
Max-Pooling 2D	–	(2, 2)	–
Convolution 2D	64	(3, 3)	ReLU
Max-Pooling 2D	–	(2, 2)	–
Convolution 2D	128	(3, 3)	ReLU
Max-Pooling 2D	–	(2, 2)	–
Convolution 2D	128	(3, 3)	ReLU
Global Average Pooling 2D	–	–	–

**Table 2:** Dataset division.

Set	Normal	Abnormal
Train	214	632
Val	33	83
Test	90	279

**Table 3:** Performance evaluation of MIL architectures for classification of cervical histopathology. LR: Learning Rate, PS: Instance/Patch Size. Number of parameters for the networks are given in the brackets.

Network	LR	PS	AUC	ACC	Recall	Precision	F1
EMILA (312354)	0.0001	344	0.7363	0.7588	0.9928	0.7610	0.8616
	0.0005	258	0.7526	**0.7642**	**0.9964**	**0.7637**	**0.8647**
	0.001	172	0.7490	**0.7642**	**0.9964**	**0.7637**	**0.8647**
	0.0005	129	**0.7562**	0.7615	0.9892	0.7645	0.8625
	0.0005	86	0.6176	0.7480	0.9749	0.7598	0.8540
EMIL_M (279073)	0.001	344	0.8363	0.8347	0.9176	0.8707	0.8935
	0.001	258	0.8316	0.8320	0.9032	**0.8780**	0.8905
	0.001	172	**0.8423**	0.8347	0.9104	0.8759	0.8928
	0.000005	129	0.8183	**0.8374**	**0.9427**	0.8567	**0.8976**
	0.0005	86	0.8248	0.8293	0.9355	0.8529	0.8923
IMIL_A (312354)	0.001	344	0.8368	0.8320	0.9176	0.8678	0.8920
	0.00005	258	0.8307	0.8347	**0.9462**	0.8516	0.8964
	0.001	172	**0.8413**	0.8320	0.9032	0.8780	0.8905
	0.00005	129	0.8391	**0.8401**	0.9068	**0.8846**	0.8956
	0.00005	86	0.8361	0.8374	0.9427	0.8567	**0.8976**
IMIL_M (279073)	0.001	344	0.8364	0.8320	0.9176	0.8678	0.8920
	0.0005	258	0.8268	0.8320	0.9319	0.8581	0.8935
	0.001	172	**0.8383**	0.8320	0.9068	**0.8754**	0.8908
	0.00005	129	0.8255	0.8347	**0.9534**	0.8471	**0.8971**
	0.00001	86	0.8213	**0.8374**	0.9355	0.8614	0.8969
ResNet-50 (23589761)	0.001	–	0.7562	0.7209	0.7921	0.8308	0.8110

**Table 4:** Variation of AUC based on $\lambda_{L0}$ and $\lambda_{\text{mer}}$ regularizer weights. PS: Instance/Patch Size.

PS	$\lambda_{L0}\to$ $\lambda_{\text{mer}↓}$	0.000	0.100	0.010	0.005	0.001
344	0.000	0.8367	0.8282	0.8208	**0.8368**	0.8352
	0.100	0.8306	0.8310	0.8354	0.8351	0.8361
	0.010	0.8303	0.8297	0.8319	0.8284	0.8271
	0.005	0.8335	0.8288	0.8299	0.8276	0.8315
	0.001	0.8331	0.8278	0.8245	0.8343	0.8343
258	0.000	0.8332	0.8231	0.8172	0.8292	0.8098
	0.100	0.8344	0.8260	0.8292	0.8282	0.8183
	0.010	0.8214	0.8335	0.8201	**0.8346**	0.8266
	0.005	0.8234	0.8293	0.8320	0.8077	0.7926
	0.001	0.8184	0.8330	0.8038	0.8276	0.8333
172	0.000	0.8394	0.8345	0.8281	0.8400	0.8401
	0.100	0.8358	0.8350	0.8366	0.8380	**0.8413**
	0.010	0.8352	0.8201	0.8367	0.8333	0.8311
	0.005	0.8379	0.8349	0.8357	0.8327	0.8380
	0.001	0.8378	0.8337	0.8309	0.8378	0.8382
129	0.000	0.8372	0.8341	0.8204	0.8360	0.8364
	0.100	0.8397	0.8325	0.8360	0.8358	0.8415
	0.010	0.8363	0.8362	0.8355	0.8414	0.8421
	0.005	0.8332	0.8416	0.8371	0.8209	0.8306
	0.001	**0.8424**	0.8365	0.8368	0.8348	0.8406
86	0.000	0.8291	0.8317	0.8291	0.8258	0.8122
	0.100	0.8276	0.8325	0.8367	0.8205	0.8221
	0.010	0.8207	0.8249	0.8312	0.8043	0.8028
	0.005	0.8373	0.8290	0.8020	**0.8425**	0.8303
	0.001	0.8224	0.8389	0.8264	0.8387	0.8385

**Table 5:** Performance evaluation of Regularized Sparsemax IMIL architectures for classification of cervical histopathology. $\lambda_{LO}$: weight for L0 regularization, $\lambda_{\text{mer}}$; weight for MER regularization, PS: Instance/Patch Size.

Parameter			Performance			
PS	$\lambda_{L0}$	$\lambda_{\text{mer}}$	ACC	Recall	Precision	F1
344	0.005	0.000	0.8401	0.9211	0.8741	0.8970
258	0.005	0.010	0.8374	0.9176	0.8737	0.8951
172	0.001	0.100	0.8401	0.9247	0.8716	0.8974
129	0.000	0.001	0.8455	0.9211	0.8801	0.9002
86	0.005	0.005	0.8401	0.9319	0.8667	0.8981

## Footnotes

1 Mathematically, Softmax has full support.
